# Infants hospitalized for Bordetella pertussis infection commonly have respiratory viral coinfections

**DOI:** 10.1186/s12879-017-2567-6

**Published:** 2017-07-12

**Authors:** A. Frassanito, R. Nenna, A. Nicolai, A. Pierangeli, A. E. Tozzi, P. Stefanelli, R. Carsetti, C. Concato, I. Schiavoni, F. Midulla, the Pertussis study group, G. Di Mattia, E. Pandolfi, F. Gesualdo, E. Agricola, L. Russo, B. Ferretti, I. Campagna, A. Villani, M. V. Gonfiantini, V. Marcellini, V. Spuri Vennarucci, G. Buttinelli, G. Fedele

**Affiliations:** 1 Department of Pediatrics, “Sapienza” University of Rome, V.le Regina Elena 324, 00161 Rome, Italy; 2 Molecular Medicine Department, “Sapienza” University of Rome, Rome, Italy; 3 Multifactorial Disease and Complex Phenotype Research Area, Bambino Gesù Children’s Hospital, Rome, Italy; 4 Department of Infectious, Parasitic & Immunomediated Disease, Istituto Superiore di Sanità, Rome, Italy; 5 Immunology Unit, Immunology and Pharmacal Therapy Area, Bambino Gesù Children’s Hospital, Rome, Italy; 6 Virology Unit, Bambino Gesù Children’s Hospital, Rome, Italy

**Keywords:** Pertussis, Respiratory virus, Severity, Child

## Abstract

**Background:**

Whether viral coinfections cause more severe disease than *Bordetella pertussis* (*B. pertussis*) alone remains unclear. We compared clinical disease severity and sought clinical and demographic differences between infants with *B. pertussis* infection alone and those with respiratory viral coinfections. We also analyzed how respiratory infections were distributed during the 2 years study.

**Methods:**

We enrolled 53 infants with pertussis younger than 180 days (median age 58 days, range 17–109 days, 64.1% boys), hospitalized in the Pediatric Departments at “Sapienza” University Rome and Bambino Gesù Children’s Hospital from August 2012 to November 2014. We tested in naso-pharyngeal washings *B. pertussis* and 14 respiratory viruses with real-time reverse-transcriptase-polymerase chain reaction. Clinical data were obtained from hospital records and demographic characteristics collected using a structured questionnaire.

**Results:**

28/53 infants had *B. pertussis* alone and 25 viral coinfection: 10 human rhinovirus (9 alone and 1 in coinfection with parainfluenza virus), 3 human coronavirus, 2 respiratory syncytial virus. No differences were observed in clinical disease severity between infants with *B. pertussis* infection alone and those with coinfections. Infants with *B. pertussis* alone were younger than infants with coinfections, and less often breastfeed at admission.

**Conclusions:**

In this descriptive study, no associations between clinical severity and pertussis with or without co-infections were found.

**Trial registration:**

Policlinico Umberto I: protocol 213/14, 3085/13.02.2014, retrospectively registered.

Bambino Gesù Children’s Hospital: protocol n. RF-2010-2317709.

## Background

Pertussis (whooping cough) is a highly contagious, respiratory disease caused by *Bordetella pertussis (B. pertussis)*. The clinical symptoms of pertussis change with age, previous exposure to *B. pertussis* and immunization status. In newborns clinical manifestations may be severe. Most infants have a typical paroxysmal cough which can last more than two months [1].

Pertussis is a major cause of morbidity worldwide and of mortality in infants in developing countries. Pertussis continues as a public health concern threat given its re-emergence despite high vaccination coverage [2]. Epidemic cycles reoccur every 2 to 5 years and 2015 has witnessed the worst outbreak in the past 70 years [3].

Although ample evidence confirms coinfections between *B. pertussis* and other pathogens, especially viruses, the role of coinfections remains debated [4–6]. Most mixed infections probably arise accidentally and whether they cause more severe disease than *B. pertussis* alone remains unclear [7–14]. Extending current knowledge on virus coinfections would make it easier to care for infants with pertussis.

We designed this study to compare clinical disease severity in infants with *B. pertussis* infection alone and those with *B. pertussis* and viral coinfections hospitalized in two Italian centers over two years. We also analyzed how respiratory infections and pertussis cases were distributed during the two years study. As primary outcome measures we assigned each infant a clinical severity score and assessed length of hospitalization. As an experimental approach to provide reliable data on lower respiratory virus infections we used an extended respiratory virus panel that can detect 14 respiratory viral targets with real-time reverse-transcriptase-polymerase chain reaction (RT-PCR) assay.

## Methods

### Patients

In a longitudinal double-center study, we enrolled 53 consecutive infants with pertussis younger than 180 days hospitalized from August 2012 to November 2014 at the Pediatric Departments “Sapienza” University and Bambino Gesù Children’s Hospital Rome. Eligible children had to have a nasopharyngeal specimen that tested positive for *B. pertussis*, be younger than 180 days, be unvaccinated against pertussis and have at least one of the following symptoms: cough lasting more than 5 days, paroxysmal cough, apnea or cyanosis and post-cough vomit.

At admission all infants underwent a nasopharyngeal washing obtained by instilling 3 ml of sterile saline into each nostril and collected with a syringe. All samples were delivered within two hours to the Department of Infectious, Parasitic & Immune-mediated Diseases at the Istituto Superiore di Sanità (Rome) for *B. pertussis* detection and to the Molecular Medicine Department (“Sapienza” University Rome Virology Laboratory) for virus detection. We considered children with *B. pertussis* infection, those with PCR positive results.

As primary outcome measures, we calculated at admission a clinical score and days of hospitalization. The clinical severity score ranged from 0 to 8, according to respiratory rate (< 45/min = 0, 45–59/min = 1, > 60/min = 2), arterial oxygen saturation in room air (> 95% = 0, 90–94% = 1, < 90% = 2), retractions (none = 0, present = 1, present + nasal flare = 2) and food intake requirement (normal = 0, reduced = 1, intravenous fluid = 2), as previously described [10]. Children’s parents were administered a structured questionnaire seeking demographic data. Demographic variables evaluated included gender, age at admission (in days), gestational age, birth weight, type of delivery, breastfeeding history, presence of siblings, number of cohabitants, presence of smoking cohabitants and cohabitants with concomitant respiratory symptoms. As secondary outcome measures, we searched the clinical records for data on the following clinical variables: heart rate, respiratory rate, arterial oxygen saturation in room air, retractions, oxygen-therapy, fluid therapy, episodes of paroxysmal cough, cyanosis, apnea, post-cough vomit, fever (body temperature > 37.5 °C), presence of skin petechiae, conjunctival hemorrhage and complications (hypoxia, bradycardia, pneumonia, gastroenteritis, thrombocytopenia, urinary tract infection, anemia, dehydration, feeding difficulties, transient hypertension). Laboratory variables investigated were white blood-cell count (WBC), lymphocyte count and C-reactive protein (CRP). A chest X-ray (CXR) obtained at hospitalization was evaluated blindly by a radiologist for consolidations.

Before infants were enrolled, all children’s parents agreed and gave written informed consent to participate in the study, which was approved by institutional review boards at both hospitals (Policlinico Umberto I: protocol 213/14, 3085/13.02.2014; Bambino Gesù Children’s Hospital: protocol n. RF-2010-2317709).

#### *Bordetella pertussis* detection by RT-PCR and culture

*B. pertussis* DNA was extracted with QIAamp DNA minikit (QiaGEM, Hilden, Germania) and amplified with the “Bordetella Real-Time PCR” kit (Diagenode Diagnostics, Liège, Belgio). The assay gave binary results. For RT-PCR the SYBR Green Detection assay we used the LightCycler 2.0 system (Roche Diagnostic). Data were analyzed with LightCycler software (version 4.0, Roche Diagnostic). Only the positive samples for *B. pertussis* were cultured on charcoal agar plates (Oxoid England) containing defibrinated sheep blood at 10% and incubated at 35 °C up to 7 days and inspected daily, as previously described [13].

### Respiratory virus detection

Nasal washings were centrifuged to remove the mucus present in the sample and an aliquot was used for nucleic acid extraction using a total nucleic acid isolation kit (Roche Diagnostics, Mannheim, Germany) and an RT-PCR panel that sought 14 respiratory viruses: influenza virus A and B (IV-A/B), human coronavirus (hCoV) OC43, 229E, NL-63, HUK1, adenovirus (AV), parainfluenza virus 1–3 (PIV 1–3), human-metapneumovirus (hMPV), human-bocavirus (hBoV), respiratory syncytial virus (RSV) and human rhinovirus (hRV), as previously described [10].

### Statistical analysis

Statistical significance was analyzed with SPSS version 23.0 (SPSS Inc., Chicago, IL, USA). Data included percentages for discrete variables, median and range for continuous variable. Differences among groups were compared using non-parametric test for median comparison, Mann-Whitney test. The χ-square test was applied to analyze categorical independent variables qualitatively. *P*-values <0.05 were considered to indicate statistical significance.

## Results

Of 53 hospitalized infants (median age 58 days, range 17–109 days, 34 [64.1%] boys) with pertussis infection enrolled, 28 (median age 51.5 days, range 17–102 days, 17 boys) had *B. pertussis* alone and 25 (median age 62 days, range 22–109 days, 17 boys) had viral coinfections. Among 25 patients with a coinfection, 9 were coinfected with hRV, 3 with hCoV, 2 with RSV, 2 with influenza virus (1 IVA and 1 IVB), 1 with PIV, 1 with AV, 1 with hMPV, 1 with hBoV and 5 patients had multivirus coinfections. Of these 53 patients, 3 had a gestational age lower than 37 weeks. A total of 4 children (3 Pertussis alone and 1 coinfected) required pediatric intensive care admission.

**Fig. 1:**
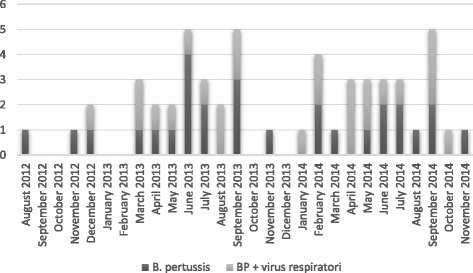
Monthly Distribution of the 53 Cases of Bordetella pertussis Infection Through the 2-Year Study

During the 2-year study *B. pertussis* infection alone was detected mainly during the summer whereas coinfections were equally distributed throughout the year (Fig. 1).

Clinical severity score and days hospitalization were similar in children with *B. pertussis* infection alone and those with *B. pertussis* and viral coinfection (Table 1).

**Table 1:** Days Hospitalization and Clinical Severity Scores in the Two Study Groups

Variables used to score clinical severity	Pertussis (*N* = 28 infants)	Pertussis + Virus (*N* = 25 infants)	*P* Values
Hospitalization stay in days	7 (1–41)	7 (2–36)	0.532^*^
Severity score median (range)	1 (0–7)	1 (0–5)	0.279^†^
Heart rate in beats per minute median (range)	145 (100–175)	140 (120–175)	0.235^†^
Respiratory rate in breaths per minute median (range)	39 (27–70)	37 (27–60)	0.702^†^
Oxygen saturation median (range)	99% (85–100%)	98% (82–100%)	0.524^†^
Retractions	7/23 (30.4%)	4/22 (18.1%)	0.602^*^
Oxygen-therapy	8/23 (34.8%)	6/22 (27.3%)	0.413^*^
Fluid therapy	7/23 (30.4%)	2/22 (9.1%)	0.077^*^

^*^by χ-square test, ^†^ by Mann-Whitney test

The questionnaire indicated that infants with *B. pertussis* alone were younger than infants with coinfections (*p* = 0.044 by Mann-Whitney test). Questionnaire answers also showed that infants with *B. pertussis* alone were less often breastfeed at admission than infants with coinfections (*p* = 0.048 by χ-square test) and for a shorter time (*p* = 0.004 by Mann-Whitney test). Finally, infants with coinfections more frequently had a higher number of cohabitants though not significantly (*p* = 0.076 by χ-square test).

The two groups had no significant differences for other demographic, clinical, laboratory and radiological data (Tables 2, 3 and 4).

**Table 2:** Demographic Variables in the Two Study Groups

Demographic variables	Pertussis (*N* = 28)	Pertussis + Virus (*N* = 25)	*P* Values
Boys	17 (60.7%)	17 (68%)	0.396^*^
Median age at hospitalization in days (range)	51.5 (17–102)	62 (22–109)	0.044^†^
Gestational age in week*s* (range)	39 (35–41)	39 (37–41)	0.105^†^
Birth weight in Kg (range)	3.345 (1.940–4.450)	3.250 (2.500–4.100)	0.618^†^
Cesarean section	10 (35.7%)	8 (32%)	0.503^*^
Breastfeeding at recovery	13 (46.4%)	19 (76%)	0.048^*^
Months breastfeeding	1.02 (0–2.14)	1.91 (0.27–3.58)	0.004^†^
Presence of siblings	16 (57.1%)	19 (76%)	0.123^*^
Number of cohabitants ≥4	19 (67.9%)	22 (88%)	0.076^*^
Passive smoke	10 (35.7%)	11 (44%)	0.798^*^
Co-habitants with respiratory symptoms	21 (75%)	22 (88%)	0.197^*^

^*^by χ-square test, ^†^ by Mann-Whitney test

**Table 3:** Clinical differences in the Two Study Groups

Clinical variables	Pertussis (*N* = 28)	Pertussis + Virus (*N* = 25)	*P* Values^*^
Duration of symptoms before hospitalization (mean ± SD)	11.4 ± 7.8	13.1 ± 9.9	0.515
Paroxysmal cough	24 (85.7%)	19 (76%)	0.291
Cyanosis	16 (57.1%)	14 (56%)	0.576
Apnea	24 (85.9%)	20 (80%)	0.425
Post-cough vomit	15 (57.6%)	14 (56%)	0.540
Fever: *T* > 37.5 °C	3 (10.7%)	5 (20%)	0.288
Skin petechiae	1 (3.6%)	4 (16%)	0.142
Conjunctival hemorrhage	0	3 (12%)	0.098
Complications^a^	10 (35.7%)	6 (24%)	0.662

^*^by χ-square test

^a^hypoxia, bradycardia, pneumonia, gastroenteritis, thrombocytopenia, urinary tract infection, anemia, dehydration, feeding difficulties, transient hypertension

**Table 4:** Laboratory and Radiological Variables in the Two Study Groups

Laboratory and radiological variables	Pertussis (*N* = 28)	Pertussis + Virus (*N* = 25)	*P* Values
White blood cells (× 10^3^ cells/μl) median (range)	15.38 (2.31–37.48)	17.04 (5.69–40.84)	0.110^†^
Lymphocytes (× 10^3^ cells/μl) median (range)	8.31 (0.1–17.88)	6.89 (0.74–35.42)	0.272^†^
WBC% Lymphocytes median (range)	61.9% (31.3–81.6%)	61% (19.3–80.1%)	0.427^†^
C-reactive protein (mg/dL) median (range)	0.05 (0–3.6)	0.05 (0.03–5.06)	0.461^†^
Consolidations on chest X-ray	4/16 (25%)	0/12	0.084^*^

^*^by χ-square test, †by Mann-Whitney test

## Discussion

In this descriptive study investigating the clinical data for 53 infants younger than 180 days hospitalized with pertussis, no associations between clinical severity and pertussis with or without co-infections were found.

As many as 25/53 (47%) of the infants hospitalized with pertussis over the two years were coinfected with other respiratory viruses. A distinctive point is that because we analyzed a wide virus battery and did so in infants hospitalized continuously in two centers over two years we feel confident that our study provides reliable data on the distribution of respiratory infections. Few studies found *B. pertussis* cases in infants with respiratory pathogens. For example, Piedra et al., identified by RT-PCR only 4 *B. pertussis* cases in 2068 patients with respiratory pathogens and all these infants were younger than 6 months. In 3 of the 4 infants with *B. pertussis* RT-PCR identified a second respiratory pathogen: 2 had an hRV and 1 child an hCoV coinfection [8]. In a similar study, Korppi et al., showed in a naso-pharyngeal aspirate by RT-PCR *B. pertussis* infection in 7 on 9 of patients hospitalized for RSV infection [15]. Our lower percentage of coinfections reflects the analysis of a larger series of children with *B. pertussis* than the other two studies.

As well as extending current epidemiological knowledge on respiratory pathogens, we confirmed that pertussis in Italy arises mainly during the summer months, whereas respiratory virus coinfections are equally distributed over the year [10]. Virological analysis showed that the most frequent virus observed was hRV (36%) and only 2 infants had RSV (8%). We presume that the distribution of coinfections reflects the observation that hRV epidemiology maintains a steady epidemic curve throughout the year and hRV can be detected also in asymptomatic children. In fact in a Netherlands study conducted in recent years Wildenbeest JG et al., found hRV in 25% of asymptomatic children [16].

A new finding in this study is the lack of differences in clinical disease severity (measured as clinical severity score and days hospitalization) in infants with *B. pertussis* infection alone and those with coinfections. Although no other published studies have tested 14 respiratory viruses in infants with pertussis, Nuolivirta et al., studied 142 infants aged less than 180 days hospitalized for bronchiolitis who underwent a nasopharyngeal aspirate to detect only 7 respiratory viruses and *B. pertussis* by RT-PCR. *B. Pertussis* involvement was found in 12 of 142 (8.5%) infants hospitalized for bronchiolitis and, of these, 8 were in coinfection with RSV. They found no differences in clinical findings, days hospitalization and breastfeeding at admission in patients with respiratory viruses alone than in patients with respiratory viruses in coinfections with *B. pertussis* [17]. Schnoeller et al. showed that respiratory infection of neonatal mice with an attenuated *B. pertussis* can protect against RSV-induced disease in adult life [18, 19]. In a human model Schiavoni et al. demonstrated that an attenuated *B. pertussis* rescues the immune functions of RSV infected human dendritic cells by promoting a protective Th1/Th17 responses. Self-limiting respiratory infections with attenuated bacteria or commensal microbes may have a beneficial effect limiting potentially lethal diseases caused by respiratory viruses in infants [20].

When we evaluated the secondary endpoints in our study, infants with *B. pertussis* alone were younger, less often breastfeed and breastfeed for a shorter time than infants with coinfections. Similarly, in a previous study from our group, comparing infants with *B. pertussis* and those with bronchiolitis, we found that the percentage of breastfed infants at hospitalization was lower in infants with *B. pertussis* than in those with RSV bronchiolitis [13]. This finding is difficult to explain. We always strongly encourage breastfeeding in infants because it has well-known immunological and nutritional advantages [21]. In a recent large multicenter study, on pertussis-associated pneumonia in children from low- and middle-income countries, pertussis-positive cases were more likely to have never been breastfed compared with controls [22]. Our finding might reflect mother-to-child transmission of respiratory infections during breastfeeding.

Our study has limitations: small sample size and our failure to compare *B. pertussis* infection with viral coinfections by single respiratory viruses.

## Conclusions

In conclusion, about one third of infants with *B. pertussis* may have a respiratory viral coinfection. These undetected coinfections seem to leave the clinical severity of pertussis in infants unchanged.

## Abbreviations

AV: Adenovirus
B. Pertussis: Bordetella Pertussis
CRP: C-reactive protein
CXR: Chest X-ray
hBoV: Human-bocavirus
hCoV OC43, 229E, NL-63, HUK1: Human coronavirus OC43, 229E, NL-63, HUK1
hMPV: Human-metapneumovirus
hRV: Human rhinovirus
IV –A/B: Influenza virus A and B
PIV 1–3: Parainfluenza 1–3
RSV: Respiratory syncytial virus
RT-PCR: Real-time reverse-transcriptase-polymerase chain reaction
T: Temperature
WBC: White blood cell
